# Abundant Harvest and Fishing for Trouble

**DOI:** 10.3201/eid1612.AC1612

**Published:** 2010-12

**Authors:** Polyxeni Potter

**Affiliations:** Author affiliation: Centers for Disease Control and Prevention, Atlanta, Georgia, USA

**Keywords:** Art science connection, emerging infectious diseases, art and medicine, fish, aquaculture, Allegory of Water, Jacopo Bassano, Renaissance masters, zoonotic infections, about the cover

**Figure:**
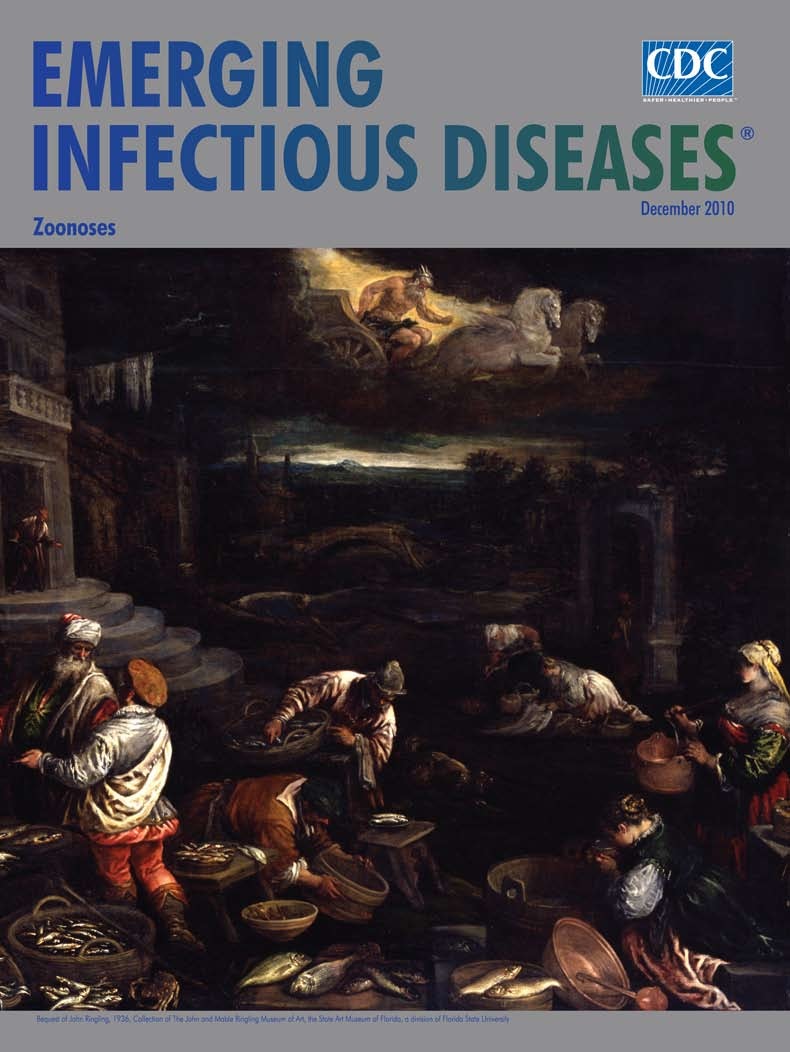
**Jacopo Bassano (c. 1510–1592) *Allegory of Water* (16th century) Oil on canvas (139.7 cm × 180.3 cm)** Bequest of John Ringling, 1936, Collection of The John and Mable Ringling Museum of Art, the State Art Museum of Florida, a division of Florida State University

“Ah, Jacopo, if you had my drawing and I had your color, I would defy the devil himself to enable Titian, Raphael, and the rest to make any show beside us,” exclaimed Tintoretto as he discussed contemporary painting with his friend Jacopo da Ponte, known as Bassano for his hometown Bassano del Grappa, near Venice. At the foot of the Alps and surrounded by chestnut woods, this small town once on the crossroads between Italy and Germany, held a special fascination, compounded over the years by aging frescoed walls and other relics of a vibrant arts community preferred by this artist over the opulent court of Emperor Rudolph, where he was invited to live.

Except for traveling to Venice to study under Veronese and later to paint in the Ducal Palace, Bassano lived and worked in Bassano del Grappa all his life, well-loved, especially by the poorest residents, who benefited from his generosity. This attachment to the local scene was perhaps why he was slighted by Vasari, who in his Lives of the Artists, only mentioned him in passing. His paintings were “very beautiful,” the famed artist and biographer deigned, “dispersed throughout Venice, and they are held in high esteem—especially the little works with animals of all kinds.” Ever so briefly, Vasari captured a specialty of Bassano’s thriving Venetian workshop: biblical themes treated as genre in domestic scenes with animals.

Least known of the 16th-century masters, Jacopo Bassano was the son of one artist and the father of four. He received his early lessons at home from his father, Francesco the Elder, a provincial painter, and retained some attachment to these humble roots, despite his grasp of art developments of his day, among them mannerism and the engravings of Dürer and Parmigianino. He led the Bassano School—a family of artists, including in addition to his sons, his son-in-law, grandson, and great-grandson—with a distinctive style and considerable output. So many used the name Bassano that distinguishing the work of individual members and admirers became a challenge for historians.

“During my voyage to Bassano,” wrote Giovanni Tiepolo in the 18th century about *St. Valentine Baptizing St. Lucia*, “I saw a miracle—a black cloak which seemed to be pure white.” Tiepolo was taken by the luminosity of color, another hallmark of Bassano’s work. Using fewer colors than his contemporaries and keeping them pure, he made them shine “like a beetle’s wing,” and he created light by deepening the shadows for contrast. His mature style, in the tradition of the High Renaissance in Venice and northern Italy, was influenced by Titian, his older contemporary and friend, whose portrait featured in *Purification of the Temple*. Great mannerist El Greco, who was in Venice in 1567 to 1570, studied the work of Bassano, as well as Tintoretto and Veronese.

Well-known in Europe during his lifetime, Bassano embraced change. In his later years, he expanded on Titian’s influence to create his personal bent—a rustic brushwork and dramatically lit night scenes. In painting *An Allegory of Water*, on this month’s cover, as was the practice in the Bassano workshop, Jacopo was probably assisted by one of his sons, likely Francesco. This type of pastoral scene, filled with still-life details and figures, usually animals around peasants engaged in everyday activities, was a novelty invented by the father–son Bassano team.

Part of a series, this painting masterfully invokes a popular subject, the physical elements water, fire, earth, and air, believed since antiquity to make up the world. Symbolism associated with them and their correspondence to internal human states and emotions, the humors, held great fascination then, as they do today, even though science has added many more elements to the original four.

Allegories, a form of metaphor common in literary works, are also an artistic device used for visual symbolic representation. By 1600, Venice was a major center of the print industry, and the rediscovery of classical texts provided an inexhaustible supply of subjects from ancient philosophy, history, and literature. *An Allegory of Water* shows Neptune, none other than Poseidon, in his chariot, gliding above the proceedings—a take from Greek mythology used to elucidate the virtues of one of the elements.

Water is the element associated with unconsciousness; darkness of night; and the moon’s monthly cycles, which control ocean tides. This element’s unique property is that, although it does not have its own specific form, it can take that of what surrounds it. Through its humidity and fluidity, water symbolizes the dissolution of form into a mass of possibilities.

Bassano uses a fish market to show the abundance that comes from the sea. The market is being set up on the waterfront just before dawn, an opportunity for a nocturnal presentation. The vendors, expertly highlighted, display a variety of seafood, while other activities involving water are taking place around them. On the left, two men are negotiating the cost of fresh catch. On the right, a woman is drinking at the well; a second one carries a bucket; while two more, near the water banks, are laundering clothes. In the background, a boat takes off.

The iconography, a mélange of realistic, biblical, and mythologic subjects, is animated by the give and take of ordinary people. The rich harvest of fish and brisk activity so early in the day, the backdrop of classical ruins, and divine presence signal that all is well in this community so heavily reliant on water. As long as Neptune is not angry and the waters flow calm, the abundant catch will feed the multitudes.

So it was, 400 years before overfishing, globalization, and aquaculture altered the bounties of the sea. Bassano’s water idyll and its harvest benefiting the local populace is not just a Renaissance tribute to the physical elements and their allegorical presence in human lives. It is also a record of a local industry long gone. Even in Bassano’s day, “local” had expanded its reach. His work was popular well beyond his hometown, certainly in Venice. And the Pearl of the Adriatic, a cluster of islands at the head of the namesake sea, was then still approachable only by water. In the 15th century, the city expanded her territory into the mainland, the *terraferma*, which included Bassano del Grappa, a source of food supply.

Tiny Venice and its astonishing success due largely to the power of water and its capacity not only to provide food but to move people and things foreshadowed today’s aquatic and periaquatic regions also heavily reliant on water harvest for ever-growing markets. Demand now as then, far exceeds local supply, prompting lucrative aquaculture in many parts of the world. But like water itself, water management is formless and dependent on local parameters.

With Neptune out of the picture, climatic and biologic tempests—among them emerging infectious diseases of fish, such as cyprinid herpesvirus 3, a highly contagious pathogen causing severe financial losses in koi and common carp culture industries worldwide—as well as foodborne and occupational zoonoses, are unleashed. Moreover, manipulation of local water resources can expose larger groups to higher levels of contagion, as in northern Vietnam, where fish-borne zoonotic trematodes have infected an estimated one million residents of the Red River Delta. Research into the basic biology of cyprinid herpesvirus 3 may lead to new treatments and control strategies for a full range of pathogenic herpesviruses. Public health research that identifies reservoirs and mechanisms of transmission of zoonotic trematodes provides the management framework for effective and efficient interventions without which fishing for the masses can overflow the boundaries of food safety.
